# Reduced Test Anxiety Among Medical Students in an Extremely Low-Stakes Examination

**DOI:** 10.1007/s40670-025-02468-8

**Published:** 2025-07-23

**Authors:** Takayasu Ito, Osamu Nomura, Katsuki Hirai, Hiroaki Ushikoshi, Takuya Saiki

**Affiliations:** 1 Center for Clinical Training and Career Development, Gifu University, Gifu, Japan; 2 Medical Education Development Center, Gifu University, Gifu, Japan; 3 Center for Regional Medicine, Gifu University, Gifu, Japan

**Keywords:** Assessment, Emotion, Medical students, Test anxiety

## Abstract

**Introduction:**

Test anxiety has a deleterious effect on the academic performance of medical students. Medical educators shifted the focus of students’ assessment from high-stakes examinations to low-stakes assessments to address the negative impacts of test anxiety on learning. Nevertheless, it is still uncertain whether low-stakes tests are an effective means of eliminating medical students’ anxiety.

**Methods:**

The study’s objective was to examine the degree to which medical students experience anxiety in the context of an extremely low-stakes test conducted in a group-based clinical reasoning class. A total of 75 students participated in the study, and their emotional responses during the clinical reasoning session and following the test session were assessed and compared using the Japanese version of the medical emotion scale.

**Results:**

The results demonstrated a decrease in self-reported anxiety levels toward the test compared with anxiety levels toward the clinical reasoning exercises conducted prior to the test (2.21 to 1.96). However, no changes were observed in the levels of other emotions.

**Discussion:**

Test anxiety experienced by medical students was found to diminish when the test was conducted in an extremely low-stakes format. This finding lends support to the efficacy of programmatic assessment from the perspective of medical students’ well-being.

**Supplementary Information:**

The online version contains supplementary material available at 10.1007/s40670-025-02468-8.

## Introduction

Test anxiety can prevent medical students from demonstrating their true competence during assessments, even when they perform well in everyday learning setting [1, 2]. In response to the challenge of test anxiety, educators and policymakers have begun to explore alternative assessment approaches that value students’ learning from their mistakes, rather than relying on high-stakes testing [3]. The paradigm shifts in medical education from high-stakes exam-based assessment to programmatic assessment can be a beneficial solution for test anxiety [4]. Programmatic assessment aims to collect evidence of learners’ competencies from a range of informative assessments to create a summative assessment by incorporating low-stakes tests into medical students’ summative assessment [5, 6]. This allows student to be evaluated on the basis of actual, optimal performance without the influence of test anxiety; however, students are still required to take numerous tests throughout their learning in medical school, regardless of whether they are low-stakes or high-stakes [7].

While the negative effects of high-stakes testing are well-documented [8], little is known about how low-stakes tests impact anxiety levels of medical trainees. Understanding this relationship is important because medical education increasingly relies on frequent low-stakes assessments to track student progress. If these assessments still cause anxiety, they may fail to achieve their intended benefits [9]. Some scholars have criticized programmatic assessment for decreasing students’ perceptions of the “stakes” involved in tests but increasing the amount of assessment time over the training process, which in turn might result in a perceived increase in the total volume of anxiety and stress [10, 11]. It is therefore imperative to examine whether medical students experience test anxiety even if the learning and test environment has extremely low stakes. This “extremely low-stakes” test can be defined as one that has negligible consequences for students’ academic progression. While there are several types and degrees of “low stakes” in assessment,” one of the key factors for students is whether the test result counts toward their final grade for the year or for graduation [10, 12]. In our specific context, the test contributed less than 5% to the overall course grade and functioned primarily as a participation check for minimizing students’ performance pressure. This low-stakes environment also can be achieved by integrating the attribute factors of group-based learning and assessment [13], which involves collaborative learning activities where students work together to solve problems, discuss cases, or complete tasks. In assessment contexts, group-based learning can take the form of team-based testing, where students are jointly responsible for answering questions or solving clinical problems [14, 15]. The collaborative nature of these environments offers opportunities for shared cognitive processing and emotional support [16]. Studies have shown that group-based assessment reduces the anxiety of examinees because of a sense of sharing the responsibility for test performance [13–16]. In addition, test formats influence student anxiety, as evidenced by the fact that medical students are less anxious in written examinations, such as short essays and multiple-choice questions (MCQs), than in clinical performance-based assessments with standardized patients, such as long cases and OSCEs [1]. While previous research has explored how low-stakes testing affects academic performance [8, 9], less is known about its impact on emotional states like anxiety. Moreover, the role of group-based learning — where students may feel a sense of shared responsibility and reduced pressure — has not been widely studied.

According to Pekrun’s CVT, anxiety tends to arise when students place a high value on test performance but feel they have limited control over the outcome [17, 18]. By examining an extremely low-stakes setting, this study explores how reduced pressure and a collaborative environment may alter these emotional responses. Therefore, we aimed to explore the impact of an extremely low-stakes test environment on test anxiety in a collaborative learning setting for medical students. We hypothesized that medical students would experience heightened anxiety during the test session, despite its low-stakes nature, due to the evaluative pressure that often accompanies the test.

## Activity

### Setting

**Fig. 1:**
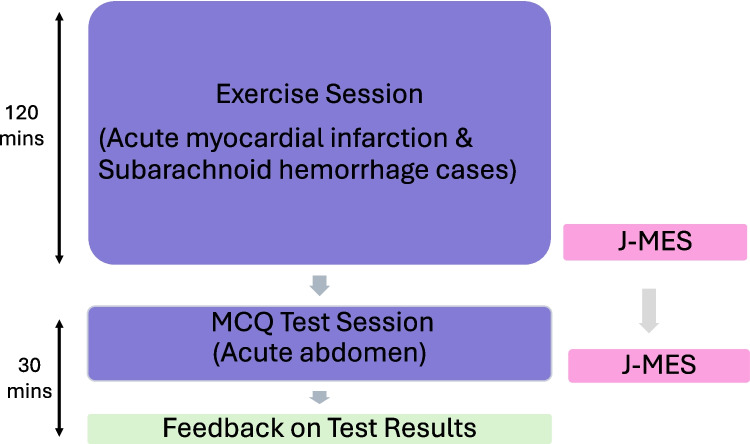
Diagram of structure for the group-based exercise and test sessions. In the first group-based exercise session, each team solved the cases of acute myocardial infarction and subarachnoid hemorrhage All the participants responded to J-MES after the exercise. In the second test session, each team solved one test case of acute abdomen. All the participants responded to J-MES after the team solved the test

This is an exploratory study examining the impact of a low-stakes examination on medical students’ perceived negative emotions such as anxiety. Seventy-five fourth-year medical students at a Japanese medical school participated in this study. In Japan, medical school spans six years, with the first four years devoted to pre-clinical education and the final two years comprising clinical clerkships. This study was embedded within the “Introduction to Clinical Medicine” course, which occurs during the final phase of the pre-clinical curriculum. The students were grouped into ten teams, with seven or eight members per group. The class schedule is illustrated in Fig. 1. Faculties or teaching assistants were assigned to each group as group tutors to facilitate the group discussions for the first exercise session. In the second test session, each student group discussed and diagnosed the scenario case, and the group collaboratively answered the MCQ test (Supplementary Material), which was also supported by the group tutors. The MCQs included seven questions asking for possible differential diagnoses and the definitive diagnosis of the abdominal pain case. The multiple-choice questions (MCQs) used in the test session were designed to align with the clinical reasoning course objectives and reflect the format of the national medical licensure examination in Japan. Questions progressed from basic recall to applied reasoning, requiring students to analyze symptoms, consider differential diagnoses, and identify the most likely conditions. The students were verbally informed and reassured by the instructor that their performance results would count only minimally toward their official grades. This arrangement was intended to provide the students with a sense of “low stakes” about the test environment.

### Measurement and Data Collection

Emotional profiles were assessed using the Japanese version of the medical emotion scale (J-MES), which has been developed and validated in accordance with Pekrun’s control-value theory (CVT) of achievement emotions [17, 18]. The J-MES comprises 20 emotion items that contain adjectives describing specific emotions, and these items are rated using a five-point Likert scale [19]. The participants responded to the J-MES immediately after each of the two sessions to determine the emotions they had experienced during the clinical reasoning activities (i.e., retrospective sampling method). The validation study of the J-MES has demonstrated that its reliability (i.e., Cronbach alpha) property for the retrospective “during” emotion items was 0.76, and its validity evidence has been assessed in various studies [18, 20–23]. Prior to the data collection phase, a sample size estimation was conducted. A previous data set from the J-MES study indicated a mean score of 2.8, a standard deviation (SD) of 1.1, and an assumed significant change of 0.5 for anxiety items. A calculation with a power of 80% and an alpha level of 5% yielded an estimated sample size of 77 people, which is a feasible target number for data collection.

### Analysis

Among the J-MES items, we analyzed the outcome-related prospective emotion items including joy, hope, anxiety, and hopelessness. The descriptive scores of the J-MES items measured for both the exercise and test sessions were summarized through calculation of mean score and SD. The independent *t*-test was used to compare J-MES scores across the exercise and test sessions. Statistical analyses were performed using IBM SPSS Statistics (version 29.0), with a significance level set at *p* < 0.05. After the statistical comparison, we also conducted a post hoc power analysis to assess the statistical power of the study.

## Results

**Fig. 2:**
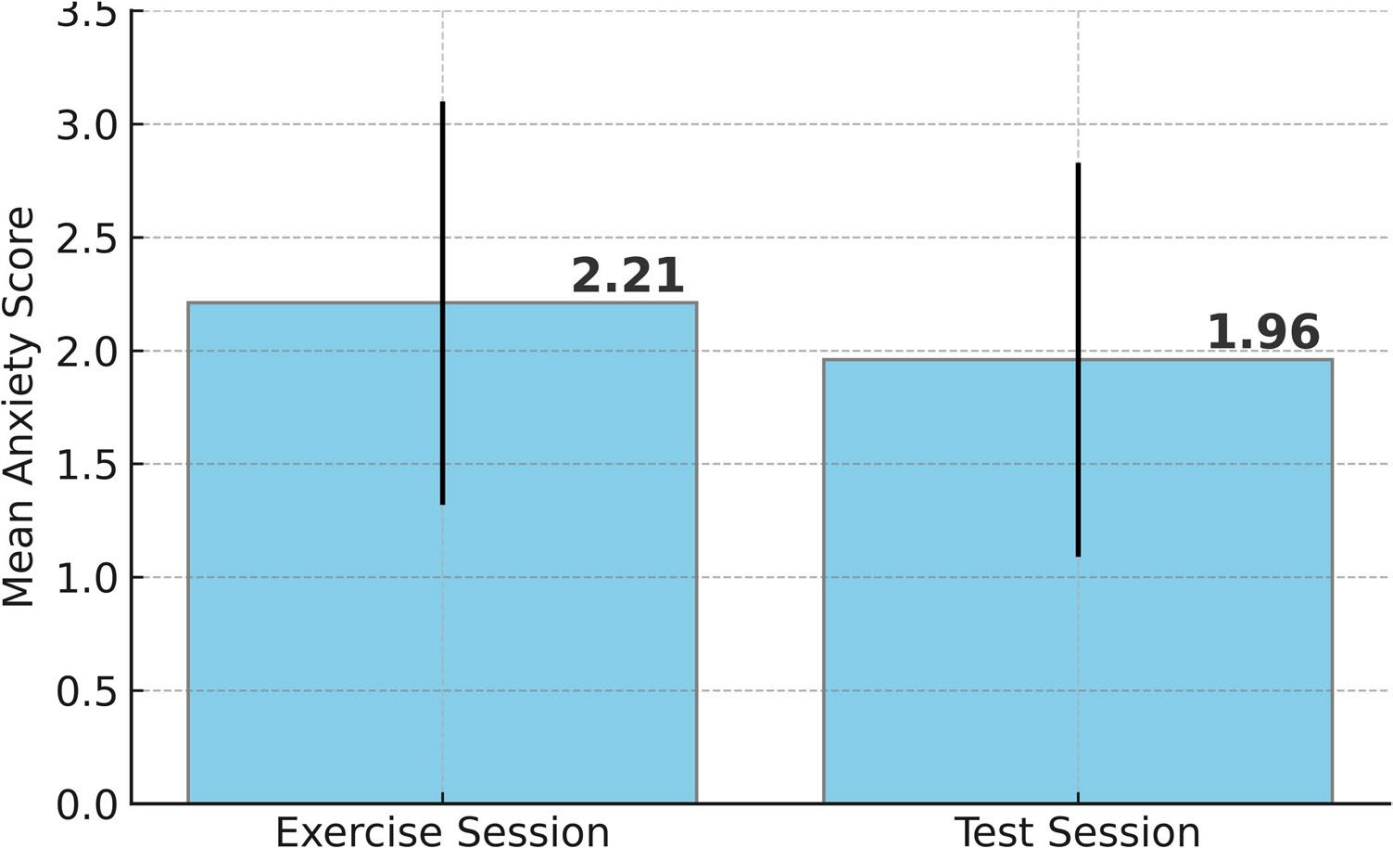
Mean self-reported anxiety scores (± SD) after the exercise session and the low-stakes test session. Anxiety levels were slightly lower during the test session (mean 1.96) compared to the exercise session (mean 2.21), although this difference did not reach statistical significance (*p* = 0.08)

Seventy-five students responded to the survey for the exercise session, and 74 students responded to that of the test session. The absence of one student response for the test session was assumed to be due to an administrative error. Table 1 presents the descriptive statistics for the J-MES items across the two sessions. The mean scores for the items pertaining to joy, hope, relaxation, and hopelessness were found to be comparable between the exercise and test sessions. Although there was no statistical significance, the mean anxiety score decreased from 2.21 (SD 0.89) to 1.96 (SD 0.87), with a *p*-value of 0.08, which was contrary to the hypothesis (Fig. 2). A post hoc power analysis of the anxiety score *t*-test revealed that the actual power and effect size were 0.41 and 0.28, respectively. The insufficient power and effect size of the statistical test may be indicative of a type II error, potentially suggesting that the insignificant difference found between the anxiety scores for the exercise and test sessions could have been significant if the sample size had been larger.

**Table 1:** Comparison of emotion items between clinical reasoning exercise and low-stakes test

	Exercise (*n*=74）	Test (*n*=75）	*p*-value	Effect size
	Mean (*SD*)	Mean (*SD*)		
Positive Activating				
Joy	3.13 (0.94)	3.27 (1.05)	0.402	0.14
Hope	2.87 (0.96)	2.82 (1.05)	0.789	0.05
Positive Deactivating				
Relaxation	2.99 (0.89)	3.09 (1.00)	0.489	0.11
Negative Activating				
Anxiety	2.21 (0.89)	1.96 (0.87)	0.080	0.28
Negative Deactivating				
Hopelessness	1.87 (0.86)	1.95 (0.87)	0.578	0.09

## Discussion

Our initial hypothesis was that medical students may experience increased anxiety toward a test even when it is low-stakes in its nature. However, the results of the current study indicate that the students’ anxiety levels during the test session trend downward compared with their anxiety levels during the practice session that took place beforehand. This unexpected outcome may have been influenced by the fact that the students perceived the test to be low-stakes and that it was conducted in a group setting. The finding thus lends support to the prevailing approach to programmatic assessment, which seeks to aggregate the performance outcomes of low-stakes examinations without unduly stressing the students. This approach also aims to provide students with prompt feedback on their performance in low-stakes examinations.

Testing is recognized as a significant source of stress for medical students, contributing to elevated levels of anxiety in academic settings [24]. In response to these findings, medical educators have sought to mitigate the adverse effects of examination-related anxiety by implementing strategies to optimize the examination environment. For example, group-based learning and the perception of interdependence between students throughout the group activity can serve to mitigate students’ anxiety [25]. This is because this pedagogical approach can facilitate psychological safety and a sense of group cohesion among the students. Therefore, instructors may consider implementing low-stakes group assessments with clearly defined expectations, supportive guidance, and immediate feedback to minimize test anxiety.

In addition, the process and mechanism for reducing their anxiety in our finding might be interpreted by applying the knowledge from cross-cultural psychology that East Asian people manage their emotions by mixing positive and negative emotions. Research in cultural psychology suggests that East Asian students often experience blended emotions—such as simultaneous negative and positive emotions — in collaborative settings. In our scenario, a slight increasing trend in the joy score was observed during the test session, which may correspond to a mixing of anxiety and joy [19, 26–28].

While the low-stakes, collaborative approach appears to have reduced negative emotions, it also presents certain limitations. The retrospective sampling method of emotional responses might have introduced recall bias. In addition, group-based testing may obscure individual performance, as more vocal or confident group members can dominate decision-making. We also acknowledge that individual emotional experiences might have varied based on group composition and interpersonal dynamics. Moreover, we did not control for pre-existing friendships or group hierarchies, which could have influenced outcomes. These limitations highlight important considerations for educators implementing group assessments. One solution is to combine group-based tests with individual reflective components, allowing for both peer collaboration and personal accountability. Statistical limitations of this study should be noted. The post hoc power analysis indicated a power level of 0.41, suggesting the sample size may have been insufficient to detect a significant difference in anxiety levels. In addition, the study’s anonymous survey design limited the ability to conduct paired *t*-test analyses, which would have allowed more precise tracking of individual emotional shifts. While these are common challenges in educational research constrained by class sizes and ethical concerns, future studies with larger cohorts and robust design would be better positioned to verify the observed trends with greater statistical confidence.

In conclusion, this study suggests that extremely low-stakes, group-based assessments may help reduce test anxiety among medical students. Even when framed as a “test,” the minimal stakes and collaborative setting appear to buffer emotional stress. While findings did not reach statistical significance, the consistent downward trend supports further exploration of these formats. These results align with the goals of programmatic assessment, emphasizing formative, low-pressure experiences that support long-term academic performance or reduce burnout, learning, and well-being. Educators may consider incorporating structured, low-stakes group assessments with clear guidance and feedback into their curricula. Future research should examine long-term emotional and academic impacts, as well as cross-cultural applicability, to strengthen the evidence base for emotionally supportive assessment practices in medical education.

## Supplementary Information

Below is the link to the electronic supplementary material.

Supplementary file 1 (DOCX 27.0 KB)

## Data Availability

The data for this study are available on reasonable request to the corresponding author.
