# Supplementary material for: Reduced Test Anxiety Among Medical Students in an Extremely Low-Stakes Examination
Source: Med Sci Educ. 2025 Jul 23;35(5):2647–52. doi: 10.1007/s40670-025-02468-8 (PMC12812151; doi:10.1007/s40670-025-02468-8)
Supplement: Supplementary file 1 — (DOCX 27.0 KB) [file 40670_2025_2468_MOESM1_ESM.docx]

**Multiple Choice Questions and Answers**

**Question 1**

**Case:** 68-year-old female
**Chief Complaint:** Abdominal pain

**What possible diseases are suspected? (Multiple answers are possible)**

- Gastrointestinal disease
- Endocrine disease
- Urological disease
- Obstetric and gynecological disease
- Cardiovascular disease

**Question 2**

**What is the most common cause of abdominal pain in the emergency room?**

- Appendicitis
- Diverticulitis
- Urinary tract stone / Ureteral colic
- Biliary colic / Cholecystitis
- Urinary tract infection

**Question 3**

**Which diseases should not be missed in cases of abdominal pain? (Multiple answers are possible)**

- Acute myocardial infarction
- Mesenteric artery thrombosis
- Bowel perforation
- Diabetic ketoacidosis
- Testicular / Ovarian torsion

**Question 4**

**Which items of the PODCAST method require urgent attention for abdominal pain? Select the top three**

- **Progression:** Worsening trend
- **Onset:** Sudden onset
- **Duration:** Within a few hours
- **Consistency:** Persistent
- **Aggravating factor:** Worsened by movement
- **Severity:** Severe pain
- **Trigger:** At rest

**Question 5**

**Chief Complaint:** Abdominal pain
**Present Illness:** Patient has experienced sudden fever and right upper abdominal pain since yesterday. Symptoms have worsened until this morning (from 5/10 to 9/10), leading to emergency transportation. Pain worsens after eating but does not radiate, and there are no changes with body position. Chills, decreased appetite, and general malaise are noted. Jaundice was identified by the emergency team.

**Past Medical History:** Osteoarthritis
**Family History:** Nothing of note
**Comorbidities:** Gallstones (under observation)
**Smoking History:** None
**Drinking History:** Wine, 400 ml per week
**Allergies:** None
**Medication:** None

**Current Findings at Visit:**
Blood pressure 140/85 mmHg, pulse rate 95 beats/min, respiratory rate 22 breaths/min
Temperature 39.0℃, SpO2 98% (room air)
Consciousness: GCS E4 V5 M6, no orientation disturbance
Conjunctiva: No anemia, Sclera: Jaundiced
Heart sounds: Regular, no murmurs
Breath sounds: Clear
Abdomen: Slight distention, soft, tenderness in the right upper quadrant, no rebound tenderness, no guarding, Murphy’s sign negative
Skin: General jaundice

**What is the most likely diagnosis at this point?**

- Acute cholecystitis
- Acute pancreatitis
- Acute appendicitis
- Acute cholangitis
- Acute hepatitis

**Question 6**

**Which findings are helpful in diagnosing this patient? (Multiple answers are possible)**

- Fever
- Right upper abdominal pain
- Decreased appetite
- General malaise
- Jaundice

**Question 7**

**What signs are expected in the advanced stage of this disease? (Multiple answers are possible)**

- Headache
- Altered consciousness
- Anemia
- Hematochezia
- Shock
